# Porous superparamagnetic activated carbon from biomass: Adsorption behaviour and regeneration performance for 2,4-dichlorophenoxyacetic acid removal

**DOI:** 10.1371/journal.pone.0353663

**Published:** 2026-07-17

**Authors:** H. Sridevi, Ramesh Vinayagam, Raja Selvaraj

**Affiliations:** Manipal Institute of Technology, Manipal Academy of Higher Education, Manipal, India; Universiti Tun Hussein Onn Malaysia, MALAYSIA

## Abstract

This research used *Tabebuia aurea* leaves to develop a porous magnetic activated carbon (TA-MAC) that helps to remove 2,4-dichlorophenoxyacetic acid. BET analysis revealed a high specific surface area of 996.87 m^2^/g, while magnetic characterization confirmed the superparamagnetic nature of the adsorbent with a saturation magnetization of 3.89 emu/g, enabling easy magnetic separation after treatment. XPS analysis verified the successful adsorption of 2,4-D through changes in surface elemental composition and functional groups. The optimum adsorption conditions were achieved at pH 2 using a TA-MAC dosage of 0.5 g/L, initial 2,4-D concentration of 50 mg/L, contact time of 120 min, temperature of 303 K, and agitation speed of 150 rpm, resulting in a maximum 2,4-D removal efficiency of 79.52%. Adsorption kinetics followed the pseudo-second order model, indicating the involvement of multiple surface interactions. The maximum adsorption capacity reached 165.18 mg/g at 303 K according to the Langmuir model. Thermodynamic evaluations further supported the physisorption nature of the interaction and confirmed its exothermic character. Additionally, the adsorbent exhibited good regeneration capacity after five cycles. TA-MAC maintained high and consistent performance, achieving over 70% 2,4-D removal efficiency in all tested real water samples, demonstrating its potential for practical applications. Collectively, these findings emphasize the efficacy of the synthesized adsorbent as a practical and sustainable solution for pollution remediation.

## 1. Introduction

2,4-dichlorophenoxyacetic acid (2,4-D) is an inexpensive and highly selective herbicide for broadleaf weeds. It finds widespread application in agricultural settings. Nevertheless, owing to its excessive water solubility and improper application, it poses a significant environmental risk. The chemical can readily leach into and persist in both groundwater and surface water [1]. Moreover, wastewater from 2,4-D production presents a serious environmental problem. If not treated correctly, this contaminated water can pollute nearby surface water sources [2]. Furthermore, research has indicated that 2,4-D is a known endocrine disruptor. The persistent exposure has been correlated to several health complications, including neurological and reproductive issues, along with potential cancer risks [3]. The existence and detrimental consequences of 2,4-D necessitate its elimination from polluted water sources, an important problem in current efforts to promote sustainability.

Due to its small size and polarity, 2,4-D is problematic for removal by standard water treatment procedures such as filtration, sedimentation, and coagulation [4]. Therefore, new and efficient treatment systems are required to prevent the accumulation of 2,4-D in water, thereby mitigating the risk of contamination in drinking water supplies. Adsorption methods are usually the most successful for eliminating 2,4-D from polluted water. It is an extremely viable technique, offering significantly high contaminant removal and a rapid process [5]. Researchers have investigated a wide range of potential adsorbent for this purpose, including activated carbon, biochar, graphene, metal-organic frameworks (MOFs), layered double hydroxides, and carbon nanotubes [6].

Activated carbon (AC) adsorbents are a popular choice because they have high surface area and are porous in nature. This structure allows them to effectively trap and remove organic pollutants such as 2,4-D. However, AC produced from plant-based biomaterials is gaining significant importance [7]. Unlike coal-based production, this method is more affordable and supports a circular economy by turning agricultural waste into a valuable product [8]. AC can be made from various parts of plants, such as stems, peels, shells, and other agricultural waste. These materials are excellent for producing AC because they are rich in carbon and contain biopolymers like cellulose. Many researchers synthesized AC from various plant-based biomaterials and utilized it for 2,4-D removal such as tomato stem [9], peanut husk [10], *Vateria indica* fruit [11], Brazil nut shell [12], peach stone [13], pineapple waste biomass [14], different parts of corn plants such as corn kernels, corn silk and corn leaves [15]. However, a significant drawback of these adsorbents is the difficulty of recovering them from water. To address this, scientists have become increasingly interested in modifying AC with magnetic nanoparticles, which significantly improves the practical applicability of the adsorbent by enabling rapid and easy separation from treated water using an external magnetic field, thereby reducing filtration requirements, operational time, and material loss during recovery and reuse. This modification makes it easier to retrieve the material after it has adsorbed pesticides from water. The magnetic separation process is also a simple, efficient, and cost-effective method [16]. Limited research exists on the 2,4-D removal using magnetic activated carbon (MAC). The few existing studies include MAC from *Sargassum siliquastrum* [17], algae [18], and yam peels [19]. This study explores the synthesis of MAC from the leaves of the *Tabebuia aurea*, also known as the Golden Trumpet Tree. While our team has previously reported on preparing AC [20] and iron oxide nanoparticles [21] from these same leaves for the removal of 2,4-D, the production of MAC from *T. aurea* leaves is a novel approach that has not been documented in the literature. Moreover, it was selected as the precursor due to its availability, carbon-rich composition, and potential to generate porous AC comparable to other commonly used agricultural biomass materials.

This research includes a thorough characterization of the synthesized MAC and an investigation into its 2,4-D removal efficiency by employing kinetic, thermodynamic, and isotherm models to understand the adsorption mechanism. Additionally, this study assessed the MAC’s reusability and its performance in treating actual water samples through spiking studies.

## 2. Materials and methods

### 2.1. Reagents and materials

2,4-D was obtained from Sisco Research Lab Pvt Ltd., and Merck, India supplied H_3_PO_4_ (85% purity), iron (II) sulfate heptahydrate (FeSO_4_·7H_2_O), NaOH and sodium bicarbonate. *T. aurea* leaves were collected from the college campus.

### 2.2. Synthesis of MAC

As reported in our previous study, *T. aurea* leaves were activated with H_3_PO_4_, resulting in AC [20] which involved pulverizing dried leaves, impregnating the powder with H_3_PO_4_ (1:2 mix), and agitating the mixture for six hours. The material was subsequently dried at 70°C, carbonized at 400°C for two hours, and purified by soaking in 1% sodium bicarbonate and rinsing to a neutral pH before being dehydrated at 100°C. The MAC was synthesized from the prepared AC using co-precipitation method, wherein AC and FeSO_4_·7H_2_O were mixed in a 2:1 ratio in 50 mL of distilled water and mixed thoroughly at ambient temperature (25–30°C) for one hour at 150 rpm. Then, 40 mL of 2N NaOH was added, and the concoction was mixed thoroughly for another 15 min. The solution was subsequently heated in a water bath, maintaining the temperature of 80°C for one hour [22]. The final product, designated as TA-MAC, was separated using a strong neodymium magnet and preserved for future use in a tightly sealed container.

### 2.3. Characterization of TA-MAC

The microstructural features of the synthesised TA-MAC were explored through field emission scanning electron microscopy (FESEM; Carl ZEISS SIGMA, Germany). The chemical nature was analysed utilising X-ray photoelectron spectroscopy (XPS; Thermo Fisher Scientific, UK). For the identification of functional groups in the TA-MAC, the Fourier transform infrared spectroscopy (FTIR; SHIMADZU-8400S, Japan) was employed. The crystalline nature was studied using X-ray diffraction (XRD; Miniflex 600, Rigaku Corporation, USA). Furthermore, the magnetic behaviour was analysed using a vibrating sample magnetometer (VSM: Versalab 3T, Quantum Design, USA) and surface area and porosity were characterized through the application of the Brunauer-Emmett-Teller (BET) apparatus (Smart Instruments, Mumbai).

### 2.4. Experimental setup for adsorption

To assess the adsorption potential of TA-MAC for 2,4-D removal, a sequence of batch trials was executed. The impact of various key parameters – including pH (2–12), adsorbent dosage (0.2–1.0 g/L), initial 2,4-D concentration (20–100 mg/L), and contact duration (up to 120 min), was systematically investigated at 303 K. TA-MAC was dosed into 100 mL of a specified 2,4-D solution concentration. The contents were then agitated for a designated period and at a precise temperature using a temperature-controlled shaker set to 150 rpm. Following each experiment, the residual concentration of 2,4-D was measured at a wavelength of 283 nm, using a UV-vis spectrophotometer (UV1900, SHIMADZU, Japan). 2,4-D removal (%) and adsorption capacity (q_e_, mg/g) were subsequently computed based on Equations (1) and (2).


$$\begin{array}{c} \text{2},\text{4}-\text{D removal }(\%)\;=\frac{\left({\text{C}}_{\text{0}}-\;{\text{C}}_{\text{f}}\right)}{{\text{C}}_{\text{0}}}\;\times \text{100} \end{array}$$ (1)

$$\begin{array}{c} \;{\text{q}}_{\text{e}}\;=\frac{\left({\text{C}}_{\text{0}}-\;{\text{C}}_{\text{e}}\right)}{m}\text{ V} \end{array}$$ (2)

C_0_, C_f_, and C_e_ represent the initial, final and equilibrium concentrations of 2,4-D, respectively. The volume of the solution is indicated by V (L), and the mass of the adsorbent is expressed as m (g).

### 2.5. Adsorption models

An extensive comprehension of adsorption isotherms and kinetics are fundamental for assessing the binding behavior of adsorbents and pollutants. To determine the optimal model to fit the available empirical data, three non-linear kinetic models were utilized: the pseudo-first order (PFO), the pseudo-second order (PSO), and the intra-particle diffusion (IPD) models (Equations 3–5).


$$\begin{array}{c} q_{t}=\;q_{e}\;(\;\text{1}-\;e^{{-k}_{\text{1}}t}) \end{array}$$ (3)

$$\begin{array}{c} q_{t}=\;\frac{\overset{\text{2}}{\underset{e}{q}}\;k_{\text{2}}\;t}{\text{1}+\;q_{e}\;k_{\text{2}}\;t} \end{array}$$ (4)

$$\begin{array}{c} q_{t}=k_{dif}t^{\text{0}.\text{5}}+C \end{array}$$ (5)

$q_{t}\;$and $q_{e}\;$represent the quantity of substances adsorbed per unit mass of adsorbent at a specific duration t and at equilibrium, respectively. These values are typically expressed in milligrams per gram (mg/g). $k_{\text{1}}\left({\text{min}}^{-\text{1}}\right)\;$is the PFO rate constant, $k_{\text{2}}(\text{g}/\text{ mg min})\;$is the PSO rate constant. $k_{dif}\;(({\text{mg}/\text{g})\text{min}}^{\text{0}.\text{5}})\;$is the IPD coefficient, which indicates the rate of diffusion within the pores of the adsorbent. C (mg/g) is a constant related to the diffusion boundary layer thickness and provides insight into the resistance to interfacial mass transfer.

A statistical analysis was performed on the adsorption data employing three distinct isotherm models: Langmuir, Freundlich, and Temkin. The Freundlich model (Eq. 6), assumes that a heterogeneous surface is formed. The Langmuir model (Eq. 7), postulates that adsorption occurs on a uniform surface, creating a monolayer where the adsorbed molecules don’t interact with one another. According to the Temkin model (Eq. 8), assumes that interactions between adsorbed molecules on a heterogeneous surface facilitate a decreasing trend in the heat of adsorption rate with a growing degree of surface coverage.


$$\begin{array}{c} q_{e}=\;K_{F}\overset{\text{1}/n}{\underset{e}{C}} \end{array}$$ (6)

$$\begin{array}{c} q_{e}=\;\frac{q_{m}\;K_{L}C_{e}}{\left(\text{1}+K_{L}C_{e}\right)} \end{array}$$ (7)

$$\begin{array}{c} q_{e}=B\;\text{ln}\left(K_{T}C_{e}\right) \end{array}$$ (8)

The following are an outline of the variables used in these models: C_e_ is the equilibrium concentration (mg/L); for the Freundlich model, K_F_ ((mg/g) (mg/L)^- 1/n^) is the Freundlich constant and n (dimensionless) is the exponent; q_m_ in the Langmuir model, represents the maximum monolayer uptake (mg/g) and K_L_ is the Langmuir constant (L/mg); and for the Temkin model, K_T_ is the Temkin constant attributed to the equilibrium binding capacity (L/mg), T denotes the temperature (K), while the ideal gas constant is represented as R (8.314 J/mol·K), and B (dimensionless) is defined as (RT/b_T_). As a function of adsorption heat, b_T_ (kJ/mol) denotes the adsorption energy factor.

Equilibrium analyses were executed at temperatures spanning from 293 to 323 K to examine the effects of temperature on 2,4-D adsorption. A non-linear Van’t Hoff plot was used to calculate the thermodynamic parameters, namely the standard enthalpy change (ΔH°) and standard entropy change (ΔS°) (Eq. 10). Equation 11 was then used to determine the standard Gibbs free energy (ΔG°).


$$\begin{array}{c} {\text{K}}_{\text{T}}\;=\;{\text{q}}_{\text{e}}/{\text{C}}_{\text{e}} \end{array}$$ (9)

$$\begin{array}{c} K_{T}=\text{exp}\left[\left(\frac{\Delta S\circ }{R}\right)-\left(\frac{\Delta H\circ }{R}\right)\frac{\text{1}}{T}\right] \end{array}$$ (10)

$$\begin{array}{c} \Delta \text{G}\circ \;=\;-\text{RT ln }{\text{K}}_{\text{T}}\; \end{array}$$ (11)

### 2.6. Reusability and real water application

For practical and large-scale applications, the ability to regenerate and reuse an adsorbent is critical. The reusability of TA-MAC was assessed through a sequence of adsorption-desorption cycles. An eluent that was composed of a 0.1 N NaOH solution was employed. The study was conducted for five cycles under optimal conditions for 2,4-D adsorption. After each run, the adsorbent was collected by centrifugation and then mixed with the eluent for one hour at 30°C and 150 rpm. Subsequently, it was meticulously cleansed with deionised water and desiccated for three hours at 80°C prior to utilisation in the next cycle.

An adsorption study using a real water sample is essential since it offers a more precise and authentic evaluation of an adsorbent’s efficacy under real environmental conditions. Although laboratory studies with synthetic water samples are beneficial for preliminary characterisation, they fail to consider the complex interactions present in natural water. The adsorption study was performed by introducing 2,4-D into distilled water (DW), lake water (LW), well water (WW), river water (RW), agricultural runoff (AGW), and tap water (TW) under optimal conditions.

## 3. Results and discussions

### 3.1. Comprehensive characterization of TA-MAC for Adsorption Studies

#### 3.1.1. Structural characteristics and elemental profile.

**Fig 1:**
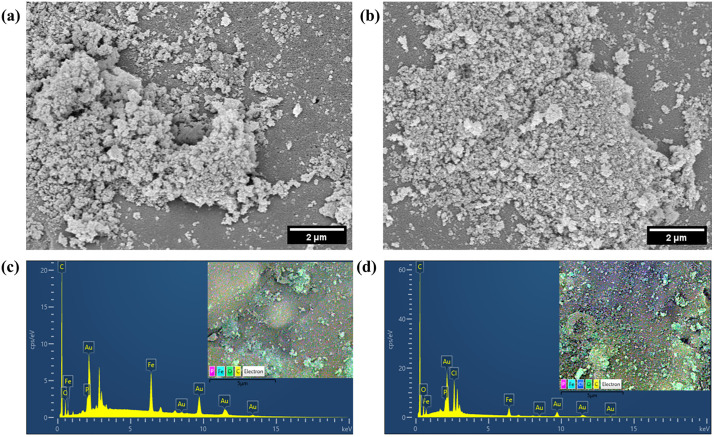
(a) prior to adsorption (b) after adsorption FESEM image. EDS picture of TA-MAC (c) before and (d) after adsorption.

The morphological characteristics of synthesised TA-MAC was obtained through FESEM image (Fig 1a). The irregular surface with a porous structure was observed to make it heterogenous surface. The abundant features of heterogeneous surfaces lead to a greater surface area, which in turn results in higher adsorptive performance. The aggregation of iron oxide nanoparticles on the surface of AC provides clear evidence of an effective bond between them and the AC surface established during the preparation [23]. A similar pattern was observed for the MAC synthesized using rice husk [24] and jackfruit peel [25]. The post-adsorption FESEM image (Fig 1b) shows structural changes caused by the aggregation of adsorbed 2,4-D molecules, indicating pore filling and an interaction among the functional groups on the TA-MAC and the 2,4-D molecules [26].

BET analysis of the synthesized TA-MAC revealed excellent textural properties, including a prominent specific surface area (SSA_BET_) of 996.87 m^2^/g, a pore volume of 0.9042 cm^3^/g, and a pore diameter of 3.63 nm (mesoporous). The pristine AC synthesized from the same precursor, as reported in our previous study, exhibited a BET surface area of 773.21 m^2^/g [20]. The comparatively higher surface area observed for the TA-MAC may be attributed to the incorporation of Fe_3_O_4_ nanoparticles, which likely prevented pore collapse during synthesis and facilitated the development of additional mesoporous structures. Importantly, this observed SSA_BET_ is superior to values reported in recent literature, such as 568 m^2^/g for MAC derived from chestnut shell waste [27], 331.5 m^2^/g for MAC derived from Sapelli wood sawdust [28], and 155.09 m^2^/g for MAC derived from orange peel [29].

EDS analysis (Fig 1c) validated the effective synthesis and elemental composition of TA-MAC, revealing it was primarily composed of carbon and oxygen, with critical amounts of iron and minor phosphorus attributed to phosphoric acid activation during the synthesis. The measured iron and oxygen content is the key indicator of successful synthesis of MAC, endorsing the integration of iron oxide nanoparticles onto the AC surface [30]. Characteristic energy peaks were witnessed at 0.27 keV for carbon, 0.5 keV for oxygen, 0.705 keV and 6.398 keV for iron, and 2.0 keV for phosphorus, while a gold peak was noted as an artifact from the image-enhancing sputtering process. After adsorption of 2,4-D, the elemental composition of the material changed (Fig 1d). Specifically, the chlorine peak at 2.621 keV serves as evidence that 2,4-D was successfully adsorbed by the TA-MAC. This is further confirmed by element mapping (Fig. 1c and 1d insets), which clearly shows chlorine’s presence post-adsorption.

#### 3.1.2. XRD and FTIR results.

**Fig 2:**
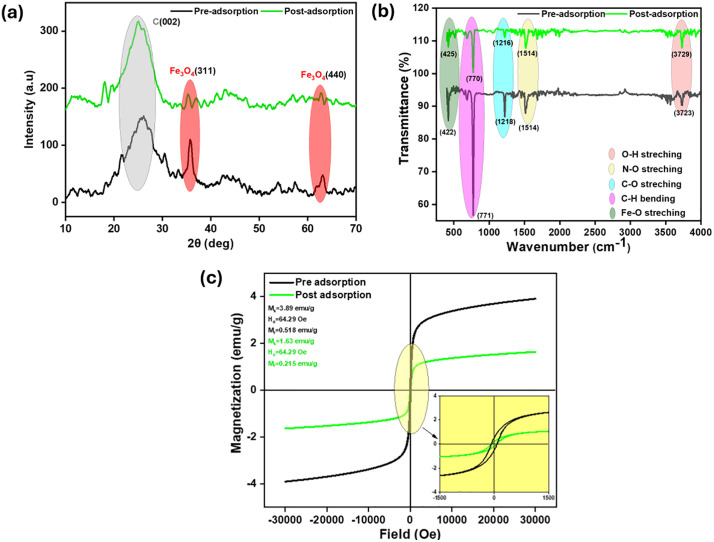
Images of (a) XRD (b) FTIR spectra (c) VSM.

As shown in **Fig 2a**, the XRD pattern for TA-MAC displayed a distinctive broad peak at about 25.93°. This broad reflection, which is associated with the carbon (002) plane, acts as evidence for the amorphous carbon structure and suggests the presence of disordered graphitic lattices in the AC matrix [31]. The appearance of peaks at 2θ of 35.77° and 63.07° are corresponds to (311) and (440) planes, indicating the presence of Fe_3_O_4_ crystallites, which are characterized by cubic spinel structures (JCPDS 19–0629) [32]. With a crystallite diameter of 29.16 nm, the incorporated Fe_3_O_4_ nanoparticles contribute to a greater TA-MAC surface area. XRD analysis performed after 2,4-D adsorption showed notable spectral changes. While the carbon matrix pattern remained largely consistent, the peak intensity associated with the Fe_3_O_4_ nanoparticles was visibly altered. This observation hints that 2,4-D molecules interact directly with the Fe_3_O_4_ nanoparticles, which leads to slight strain or lattice distortions in the Fe_3_O_4_ crystal [33].

FTIR spectra of TA-MAC for both pre- and post-adsorption processes are shown in **Fig 2b**. The pre-adsorption spectrum exhibits a subtle signal near 3723 cm^-1^, ascribed to the unbound O-H bonds found in polyphenolic compounds derived from *T.aurea* leaves [34]. In addition to these distinctive vibrations, nitrogen compounds exhibit a peak at 1514 cm^-1^, which is consistent with N-O stretching, and an absorption peak at 1218 cm^-1^ signifying C-O stretching in phenols and esters [35]. Furthermore, C-H bending in aromatic ring structures is confirmed by the prominent peak at 771 cm^-1^ [36]. The occurrence of iron oxides on the MAC was confirmed by absorption bands between 400–500 cm^-1^, particularly the absorption peak at 422 cm^-1^, which signifies the presence of Fe-O bonds [37]. Following 2,4-D adsorption, shifts were recorded in the characteristic infrared bands. The vibrational bands at 422, 771, 1218, and 3723 cm^-1^ exhibited displacements, moving to 425, 770, 1216, and 3729 cm^-1^, respectively. Moreover, there was change in the intensity of these bands, which strongly suggests molecular affinity between TA-MAC and 2,4-D molecules.

#### 3.1.3. Magnetic properties.

The magnetic property of TA-MAC was assessed using VSM analysis as displayed in Fig 2c. The magnetization curves expose a sigmoidal curve with the magnetization saturation (M_s_) of 3.89 emu/g, exhibiting the superparamagnetic behaviour with low coercivity (H_c_ = 64.29 Oe) and remanent magnetization (M_r_ = 0.518 emu/g). Moreover, the remanent ratio (M_r_/M_s_) is 0.133, which is <<1, further confirming its superparamagnetic nature [38]. The adsorption of 2,4-D molecules had a measurable effect on the TA-MAC’s magnetic properties. The decrease in saturation magnetization from 3.89 to 1.63 emu/g after 2,4-D adsorption is mainly due to the surface coverage of TA-MAC by non-magnetic 2,4-D molecules, which partially shields the magnetic domains. Furthermore, no significant phase or chemical changes in the Fe_3_O_4_ structure were observed from the XRD, FTIR, and XPS analyses after adsorption, indicating that the magnetic component remained stable during the process. Although the saturation magnetization decreased after adsorption, the adsorbent could still be effectively separated from the aqueous solution using an external magnetic field [39]. Supporting this, the remanent magnetization slightly changed (from 0.518 emu/g to 0.215 emu/g), confirming an interaction between the adsorbed molecules and the magnetic nanoparticles [33]. Furthermore, the TA-MAC sample demonstrates superior magnetic performance, achieving a higher magnetic saturation than several other reported MAC, such as 3.61 emu/g for MAC synthesized from commercial AC [40], 2.889 emu/g for MAC derived from Lonicera leaves [41], and 2.27 emu/g for MAC derived from copper pod [22].

#### 3.1.4. XPS results.

**Fig 3:**
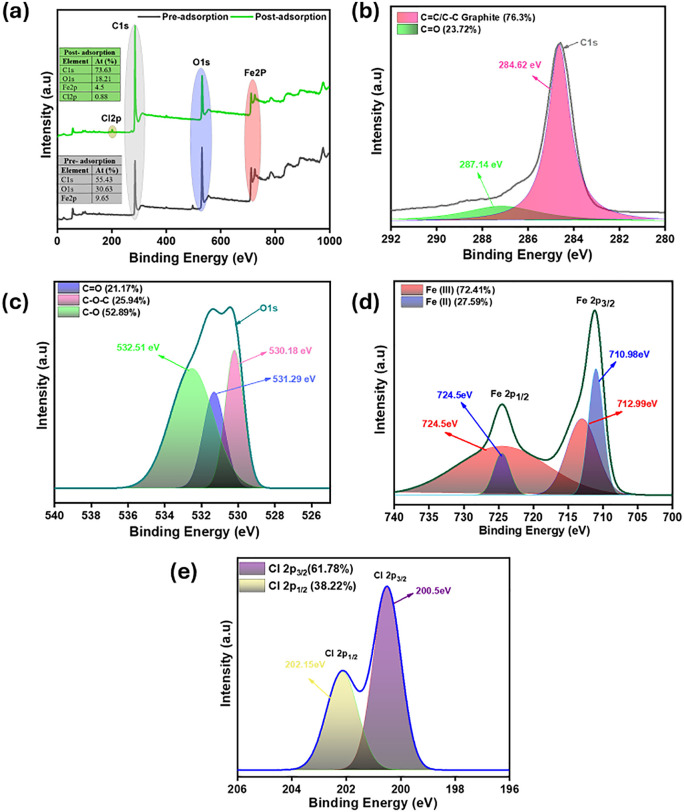
(a) XPS spectra of TA-MAC. High resolution deconvolution XPS spectra for (b) C1s (c) O1s (d) Fe2p (e) Cl2p of TA-MAC.

Analysis of the TA-MAC surface prior to the adsorption process was conducted employing X-ray photoelectron spectroscopy (XPS), as depicted in Fig 3a. The resulting spectrum confirmed the presence of key functional groups, displaying a prominent peak for Carbon (C1s) at 285 eV [42] and an oxygen signal (O1s) at 531 eV [43]. A signal for Fe2p at 711 eV was detected, verifying the magnetic nature of the TA-MAC material. Elemental composition before adsorption was quantitatively determined to be: C1s (55.43%), O1s (30.63%), Fe2p (9.65%), and P2p (0.15%). After the adsorption experiment, notable changes in elemental percentage were observed: the significant rise in the C1s signal to 73.63% is directly attributed to the carbon atoms introduced by the successful uptake of the 2,4-D compound, oxygen (O1s) decreased to 18.21%, and iron (Fe2p) dropped to 4.5%. This shift suggests that the iron oxide nanoparticles had successfully interacted with the 2,4-D molecule. The presence of a new signal for chlorine (Cl2p) at 201 eV, registering an atomic percentage of 0.88% in the post-adsorption XPS data, provides additional confirmation of the successful loading of 2,4-D onto the TA-MAC [44]. These results align with the conclusions from the EDS analysis.

XPS deconvolution was performed on the TA-MAC surface after 2,4-D adsorption to pinpoint the participating functional groups. The deconvoluted C1s spectrum in **Fig 3b** reveals two principal components, including: C-C/C = C graphitic content observed at 284.62 eV (76.3%) and C = O group identified at 287.14 eV (23.72%) resulting from an increase in carboxyl group after 2,4-D adsorption on TA-MAC through π-π interactions and hydrogen bonding [45]. The high-resolution O1s spectrum provided additional evidence supporting these findings (Fig 3c), which showed peaks for C-O-C at 530.18 eV (25.94%), C = O at 531.29 eV (21.17%), and C-O at 532.51 eV (52.89%). The Fe2p spectrum (Fig 3d) confirms the presence of both Fe(III) and Fe(II) oxidation states. Distinct peaks observed at 724.5 eV and 712.99 eV correspond to Fe(III), whereas the weaker signals at 724.5 eV and 710.98 eV were ascribed to Fe(II). Quantitative analysis reveals that Fe(III) dominates the surface composition, accounting for approximately 72.41%, while Fe(II) represents 27.59% [46,47]. The predominance of Fe(III) likely promotes the greater adsorption of 2,4-D on TA-MAC. The Cl2p spectrum (Fig 3e) shows two distinct peaks agreeing to Cl2p_3/2_ at 200.5 eV (61.78%) and Cl2p_1/2_ at 202.15 eV (38.22%), confirming the loading of organic chlorine and signifying the favorable adsorption of 2,4-D on TA-MAC [48]. Overall, the XPS findings imply that the 2,4-D adsorption on TA-MAC primarily occurs through physisorption mechanisms, aided by π–π stacking and hydrogen bonding.

### 3.2. Adsorption results

#### 3.2.1. The influence of pH.

**Fig 4:**
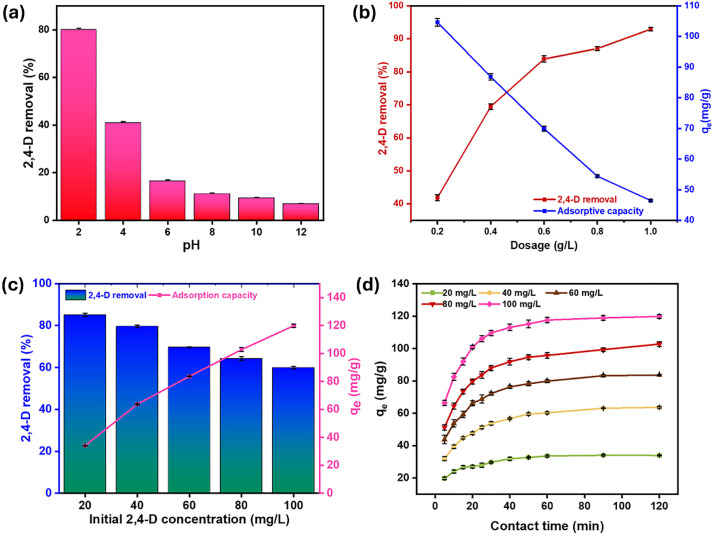
Impact of (a) pH (b) TA-MAC dosage (c) Initial 2,4-D concentration (d) Contact time on 2,4-D adsorption.

The pH dependent behavior of TA-MAC was analyzed by systematically changing the solution pH between 2 and 12 using 50 mg/L of 2,4-D concentration and 0.5 g/L of TA-MAC. The data (Fig 4a) revealed that a low pH of 2 yielded the greatest 2,4-D removal with the maximum removal efficiency of 80.04%. However, raising the solution pH triggered a sharp decline in performance, with the removal efficiency falling to 6.94% at pH 12. The maximum removal at low pH is achieved because the acidic environment enables strong electrostatic binding, hydrogen bonding, and π-π binding to work together effectively. The TA-MAC surface is positively charged below the pH_zpc_ (3.03), and the 2,4-D molecules primarily persist in its undissociated form, with only a minor anionic fraction present, given that the pH is below its pK_a_ value (2.8) [18]. This facilitates electrostatic binding between the minor fraction of anionic 2,4-D species and positively charged TA-MAC. However, removal of 2,4-D cannot be attributed solely to electrostatic attraction, since both the adsorbent surface and 2,4-D molecules exist largely in near-neutral forms. Moreover, the undissociated neutral form of 2,4-D molecules can also form hydrogen bonding between the oxygen-bearing functional group on TA-MAC. In addition to this, the neutral 2,4-D molecules are more hydrophobic and prone to interactions between non-polar regions of TA-MAC, causing π-π interaction [49]. At higher pH values, the 2,4-D deprotonates and exists predominantly as a negative ion. Since the TA-MAC surface is also highly negative above its pH_zpc_, this pH change results in powerful electrostatic repulsion, significantly reducing the TA-MAC’s potential to remove 2,4-D. Moreover, at elevated pH values, hydroxyl ions vigorously contend with the anionic 2,4-D for the remaining accessible adsorption sites on the TA-MAC surface [21]. Collectively, these effects explain the steep drop in removal efficiency.

#### 3.2.2. The influence of TA-MAC dosage.

The impact of adsorbent dosage was examined by running experiments across incremental values varying from 0.2 to 1 g/L. During these trials, a constant 2,4-D concentration (50 mg/L), a temperature of 303 K, a fixed pH of 2.0, and an agitation speed of 150 rpm were maintained. The results in **Fig 4b** demonstrate that advancing the dosage caused the percentage removal of 2,4-D to rise sharply (from 41.86% to 92.94%), attributed mainly to the rise in accessible binding sites for 2,4-D adsorption. On the contrary, the adsorption capacity declined from 104.66 to 46.47 mg/g with higher TA-MAC doses. The reduced adsorption is attributed to the saturation of the TA-MAC surface by 2,4-D molecules [50]. Adding more TA-MAC is ineffective because most pollutants are already bound. The intersection of the two data curves occurred at a dosage of 0.46 g/L, achieving 73.77% removal and the adsorption capacity of 83 mg/g. This dosage was considered to provide the best compromise between maximizing overall percentage removal and retaining a reasonable adsorption potential. Therefore, 0.5 g/L was chosen as the standard dosage for the rest of the study.

#### 3.2.3. The influence of contact duration and 2,4-D concentration.

The effect of both contact time and initial 2,4-D concentration was examined by adjusting the 2,4-D concentration across the range of 20–100 mg/L and contact time between 0–120 min. For all tests, the experimental conditions were fixed at: temperature of 303 K, agitation speed of 150 rpm, pH of 2.0, and adsorbent dosage of 0.5 g/L. The study revealed an inverse association between adsorption potential and efficiency as the initial 2,4-D concentration inclined (from 20 to 100 mg/L). Specifically, the adsorption potential rose significantly (from 34.03 to 119.85 mg/g), as depicted in **Fig 4c**, indicates strong interaction and a high concentration gradient, while the adsorption efficiency dropped sharply (from 85.1% to 40.07%) due to limited active sites [51]. As the contact duration increased, adsorption was rapid at initial stage due to high concentration gradient and abundance of active sites driven by the material’s impressive pore volume and specific surface area. Importantly, equilibrium adsorption was attained within 120 min across all tested concentrations as shown in the Fig 4d due to active site depletion with time and reducing concentration gradient [14].

#### 3.2.4 Adsorption models.

**Fig 5:**
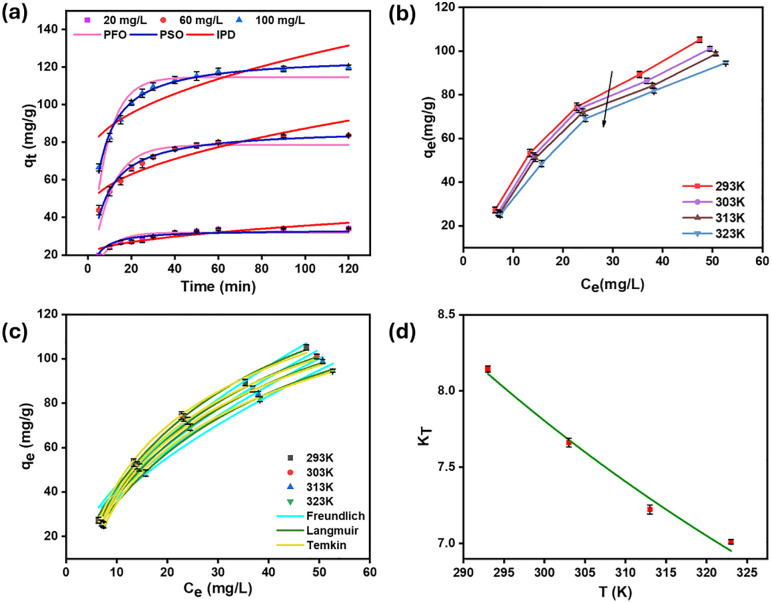
(a) Adsorption kinetic models for 2,4-D removal using TA-MAC, (b) effect of temperature on 2,4-D adsorption, (c) adsorption isotherm models for 2,4-D removal using TA-MAC, and (d) Van’t Hoff thermodynamic plot for the adsorption of 2,4-D onto TA-MAC.

It is clear from the Table 1 and Fig 5a that the PSO model offered the best fit for representing the adsorption of 2,4-D onto TA-MAC, outperforming the other two evaluated models. This is supported by the high coefficient of determination (R^2^) values (0.889, 0.979, and 0.990), the lowest reduced chi-square (χ²) values (2.647, 3.715, and 3.076), and the lowest Akaike Information Criterion (AIC) values (4.07, 23.97, and 21.89) across the 2,4-D concentrations of 20, 60, and 100 mg/L. The dominance of the PSO model implies that the rate-limiting phase in the adsorption process is chemisorption, defined as the establishment of molecular bonds between the 2,4-D and the TA-MAC surface sites. Moreover, the PSO model demonstrated exceptional reliability in predicting the adsorption behaviour. This is clearly shown by the excellent match between the predicted equilibrium adsorption capacity (q_e_) and the experimentally measured value (q_e,exp_). Across the different initial 2,4-D concentrations (20, 60, and 100 mg/L), the experimental values (34.04, 83.65, and 119.85 mg/g) were very close to the calculated values (33.37, 87.24, and 125.85 mg/g), respectively (Table 1). This strong agreement confirms that the removal of 2,4-D follows second-order kinetics, suggesting a strong attractive force between the TA-MAC material and the 2,4-D molecules. However, as the 2,4-D concentration raised, the rate constant of the PSO model dropped. This inverse relationship implies that the adsorption rate is not just about the concentration; it is ultimately governed by the limited supply of empty binding sites on the adsorbent surface [17]. The findings are consistent with prior research including MAC from Brazilian polyacrylonitrile textile fiber [52] and peanut husk [10]*.* The poor performance of the PFO and IPD models in describing the kinetics of 2,4-D adsorption onto TA-MAC is statistically confirmed by its significantly lower R^2^ values and higher χ² values when compared to the superior-fitting PSO model, as detailed in **Table 1**.

**Table 1:** Established values for the kinetic model for the adsorption of 2,4-D on TA- MAC.

2,4-D concentration (mg/L)			
Kinetic Model	20	60	100
**PFO**			
${𝐤}_{1}\;({𝐦𝐢𝐧}^{-1})$	0.14	0.112	0.103
${𝐪}_{𝐞}\;(𝐦𝐠/𝐠)$	31.99	78.54	114.63
${𝐑}^{2}$	0.777	0.860	0.807
**χ^2^**	5.34	25.57	31.86
**AIC**	18.51	42.88	45.29
**PSO**			
${𝐤}_{2}\;(𝐠/\;𝐦𝐠.𝐦𝐢𝐧)$	0.01	0.0019	0.0016
${𝐪}_{𝐞}\;\left(𝐦𝐠/𝐠\right)$ ${𝐪}_{e,exp}\;\left(𝐦𝐠/𝐠\right)$	33.37 34.04	87.24 83.65	125.85 119.85
${𝐑}^{2}$	0.889	0.979	0.990
**χ^2^**	2.647	3.715	3.076
**AIC**	4.07	23.97	21.89
**IPD**			
${𝐤}_{𝐝𝐢𝐟\;}({𝐦𝐠/𝐠)𝐦𝐢𝐧}^{0.5}$	1.59	4.41	5.55
𝐂(𝐦𝐠/𝐠)	19.77	43.23	70.63
${𝐑}^{2}$	0.832	0.843	0.754
**χ^2^**	4.033	28.70	79.81
**AIC**	16.08	46.47	57.72

As illustrated in Fig 5b, the TA-MAC’s ability to adsorb 2,4-D is negatively affected by rising temperatures, showing a clear decline in adsorption capacity as the temperature inclined from 293 to 323 K. This decrease is primarily explained by two factors: first, the elevated temperatures give the adsorbed 2,4-D molecules greater kinetic energy, making them more likely to break free and desorb from the surface, which inherently lowers the overall adsorption potential. Second, the heat may cause structural damage to the adsorbent, possibly leading to pore enlargement. This structural change reduces the material’s effective surface area, resulting in fewer available adsorption sites [43].

The research investigated the adsorption behavior across a temperature span of 293–313 K using three standard isotherm models: Langmuir, Freundlich, and Temkin. The non-linear fitting of these isotherms is presented in Fig 5c, and the determined model parameters are listed in Table 2. Drawing from the statistical analysis at 303 K, the Temkin isotherm model provides the best fit for describing the removal of 2,4-D using TA-MAC. This conclusion was drawn because Temkin yielded the highest coefficient of determination (R² = 0.996) and the lowest chi-squared value (χ² = 3.97) and the lowest Akaike Information Criterion (AIC = 34.35) compared to the Langmuir (R² = 0.989, χ² = 11.71 and AIC = 45.31) and Freundlich models (R² = 0.968, χ² = 36.53 and AIC = 57.12). The preferential fit to the Temkin model provides evidence for surface energetic heterogeneity and indirect adsorbate-adsorbate interactions. This model indicates a progressive linear decrease in the adsorption heat with increasing surface coverage. This confirms that the 2,4-D is primarily bound with TA-MAC through weak, non-covalent forces like electrostatic and Vander Waals interactions, and hydrogen bonding. Furthermore, the positive magnitude of b_T_ suggests that the adsorption process is exothermic [53,54].

**Table 2:** Isotherm parameters fitted for 2,4-D removal using TA-MAC.

Temperature (K)				
**Model**	**293**	**303**	**313**	**323**
**Langmuir**				
${𝐪}_{𝐦}$ **(mg/g)**	172.04	165.18	161.36	158.61
${𝐊}_{𝐋}\;$ **(L/mg)**	0.0324	0.0316	0.0307	0.0286
${𝐑}^{2}$	0.996	0.989	0.988	0.992
χ^2^ AIC	5.41 42.93	11.71 45.31	12.63 45.43	7.62 44.41
**Freundlich**				
${𝐊}_{𝐅}$ ((mg/g) (mg/L)^- 1/n^)	11.82	10.72	10.28	9.58
**n** (dimensionless)	1.69	1.72	1.72	1.72
${𝐑}^{2}$	0.982	0.968	0.967	0.970
χ^2^	22.15	36.53	35.58	29.72
AIC	54.40	57.12	55.29	58.18
Temkin				
B (dimensionless)	38.09	37.71	36.91	35.71
${𝐊}_{𝐓}\;$(L/mg) ${𝐛}_{𝐓}\;(𝐤𝐉/𝐦𝐨𝐥)\;$	0.313 0.064	0.286 0.067	0.277 0.070	0.265 0.075
${𝐑}^{2}$	0.996	0.996	0.995	0.995
χ2 AIC	4.45 34.91	3.97 34.35	4.61 35.09	4.29 34.7

The maximum adsorption capacity, as determined by the Langmuir isotherm, was found to be inversely proportional to temperature, increasing from 158.61 to 172.04 mg/g as the temperature dropped. Other investigations into 2,4-D adsorption have reported the same phenomenon, for instance, MAC derived from Brazilian polyacrylonitrile textile fiber [52] and MAC derived from yam peels [19]. The calculated Langmuir separation factor (R_L_) values, determined using the formula R_L_ = 1/(1 + K_L_*C_o_), fall in the narrow range of 0.38 to 0.41. Since all these values lie between 0 and 1 across the studied temperatures, they confirm that the 2,4-D adsorption onto TA-MAC was favourable and effective. Furthermore, the Freundlich constant (n) values, which represent adsorption intensity, are tightly clustered between 1.69 and 1.72. As these values are all above 1, they validate the strong efficiency of the adsorption [14]. Significantly, the maximum adsorption capacity achieved by TA-MAC at 303 K (165.18 mg/g) was higher than the other magnetic adsorbents for 2,4-D removal testified by many studies in the existing literature (Table 3).

**Table 3:** Comparison of 2,4-D removal by various magnetic adsorbents.

Magnetic adsorbent	SSA (m^2^/g)	Magnetic saturation (emu/g)	Operational condition for batch study					Adsorption potential (mg/g)	Ref
			pH	Adsorbent Dose (g/L)	Temperature (K)	Contact time (min)	Initial 2,4-D concentration (mg/L)		
**Magnetic activated carbon from Brazilian polyacrylonitrile textile fiber**	–	11	6.5	0.33	288	360	20	51.10	[52]
**Magnetic cerium oxide composite**	105.2	31.79	*	0.5	298	100	100	55.7	[61]
**Algal magnetic activated carbon**	292.51	0.921	2	2	303	60	100	60.61	[18]
**Magnetic peanut husk-MOF composite**	70	4.52	3.98	1.2	298	60	30	79.2	[49]
**Magnetic activated carbon from Yam peels**	325.22	6	2	2	298	150	100	99.009	[19]
**Magnetic hydrochar from pomegranate waste**	–	3.98	2	0.2	333	24h	100	101.10	[23]
***T. aurea* leaf derived magnetic activated carbon**	996.87	3.89	2	0.5	303	120	50	165.18	This study

*Native pH

A thermodynamic study is crucial for understanding the energy profile and feasibility of the adsorption process. A strong correlation (R^2^ = 0.987) (Table 4) was observed in the nonlinear van’t Hoff plot, validating the temperature-dependent equilibrium data shown in Fig 5d. The increase in the negative ΔG° values with rising temperature demonstrates a corresponding enhancement in the thermodynamic spontaneity of the adsorption process. Moreover, the decrease in disorder at the solid-solution barrier as 2,4-D molecules link to the binding sites of TA-MAC is suggested by the negative entropy change (ΔS° = −3.57 J/mol K). The observation indicates that the enthalpy change is negative (ΔH° = −4.05 kJ/mol) further authenticates that the adsorption process is exothermic. The exothermic nature of the adsorption process indicates that lower temperatures favor 2,4-D removal, while adsorption efficiency may decrease at elevated temperatures. For large-scale applications, this suggests that the adsorbent can perform effectively under normal environmental conditions without requiring external heating, thereby reducing energy demand and operational cost. Furthermore, the ΔH° value is within the usual range (0.4 to 80 kJ/mol), it is typical of a physical adsorption mechanism [55]. This conclusion is also in good agreement with the findings from the adsorption isotherm studies.

**Table 4:** TA-MAC’s thermodynamic components for the removal of 2,4-D.

Thermodynamic model					
T (K)	ΔG° (kJ/mol)	ΔH° (kJ/mol)	ΔS° (J/mol K)	*R* ^*2*^	χ^2^
**293**	–5.11	– 4.05	–3.57	0.987	0.005
**303**	–5.13				
**313**	–5.14				
**323**	–5.23				

#### 3.2.5. Insight into adsorption mechanism.

**Fig 6:**
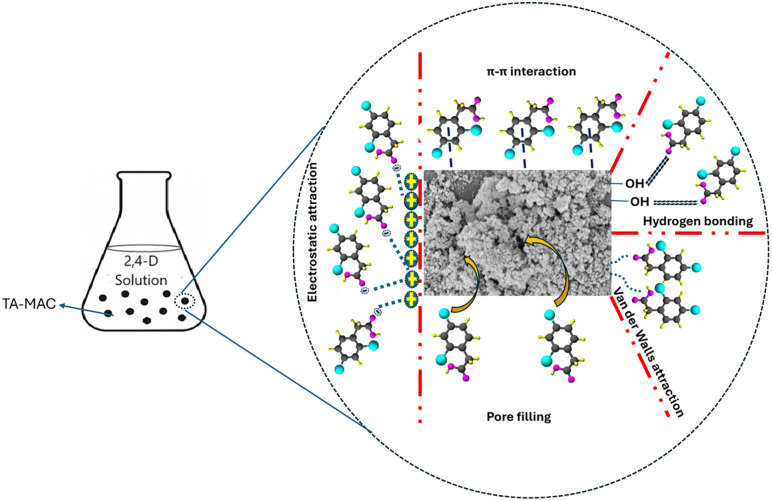
A projected mechanism of 2,4-D adsorption on TA-MAC (The 3D molecular structure of 2,4-D was generated using the ChemSketch (Freeware) 2024.1.1 platform).

The projected adsorption mechanism of 2,4-D onto TA-MAC is detailed in Fig 6. Analysis after adsorption provided several key pieces of evidence supporting this mechanism. Post-adsorption analysis strongly suggests that both pore filling and chemical interactions were involved. FESEM images showed 2,4-D molecule aggregation and structural change, indicating pore filling and interaction with the TA-MAC functional groups. EDS spectra confirmed the presence of adsorbed 2,4-D with a chlorine peak at 2.621 keV. Moreover, BET analysis confirmed the mesoporous properties of TA-MAC, with a pore size of 3.63 nm. Given 2,4-D’s molecular dimensions (1.54 × 0.56 × 0.22 nm), these findings suggest that pore-filling adsorption is favored when the adsorbate molecule is smaller than the adsorbent pore [56]. Furthermore, the higher specific surface area and improved pore structure provide additional physical adsorption sites and accessible channels for 2,4-D molecules [57]. The post-adsorption FTIR spectrum showed that several characteristic infrared bands of the TA-MAC adsorbent exhibited both shifts in frequency and attenuation of intensity at its peak following 2,4-D adsorption, providing strong evidence of molecular interactions. Specifically, the bands at 422 cm^-1^ (Fe-O), 771 cm^-1^ (C-H), 1218 cm^-1^ (C-O), and 3723 cm^-1^ (O-H) were all affected, suggesting that these functional groups, the oxygen-containing groups (Fe-O, C-O, and O-H) and the C-H group, were strongly involved in the binding process. The functional groups likely act as electron donors that engage in interaction with the aromatic ring of the 2,4-D molecule, which is functioning as an electron acceptor, establishing a significant π-π interaction as a key mechanism for the adsorption [58,59]. Furthermore, this indicates that the surface functionalization of AC with magnetic nanoparticles enhanced the 2,4-D adsorption. Post-adsorption XPS analysis of the TA-MAC confirmed the successful binding of 2,4-D, evidenced by an increase in the C1s signal and the appearance of the Cl2p band (Cl2p_3/2_ and Cl2p_1/2_ peaks) from the organochlorine molecule [48]. The decreased O1s and Fe2p signals indicate a strong interaction with the iron oxide nanoparticles. Furthermore, the rise in C-O and C = O groups on the TA-MAC surface, attributed to the 2,4-D carboxyl groups, proposes a physisorption mechanism, likely involving hydrogen bonding and π-π stacking. The Fe2p spectrum confirmed the presence and dominance of Fe(III), which is the primary contributor to the enhanced adsorption capacity for 2,4-D. Supporting this, the post-adsorption VSM analysis showed a decrease in magnetization saturation and remanent magnetization confirming an interaction between the adsorbed molecules and the magnetic nanoparticles. Maximum removal of 2,4-D is achieved at an acidic pH because the acidic environment enables strong, synergistic interactions. At pH below the pH_zpc_ of TA-MAC and the pKa of 2,4-D, the TA-MAC surface is protonated while 2,4-D exists primarily as the hydrophobic neutral form with a small anionic fraction. This condition facilitates: electrostatic binding between the positive surface and the anionic 2,4-D; hydrogen bonding between the neutral 2,4-D molecules and oxygen-bearing functional groups on TA-MAC; and π-π stacking driven by the increased hydrophobicity of the neutral 2,4-D. The simultaneous effectiveness of these three mechanisms under low pH conditions drives the high overall adsorption efficiency. The kinetic study revealed that the PSO model provided the best fit, suggesting the possible involvement of covalent interactions between the oxygen-containing functional groups on the adsorbent surface and the functional groups of 2,4-D molecules.The isotherm studies confirms that the Temkin model was the best suited model with low heat of adsorption (b_T_) confirming physisorption involving non-covalent forces like electrostatic and Vander Waals interactions, and hydrogen bonding. Based on the thermodynamic investigation (ΔH° = −4.05 kJ/mol), physisorption is confirmed as the main process. This indicates that the adsorption of 2,4-D on TA-MAC is multi-mechanistic in nature, simultaneously involving various interactions such as pore filling, electrostatic attraction, Van der Waals forces, hydrogen bonding, π-π interactions, and possible covalent bond formation between functional groups of the adsorbent and 2,4-D molecules.

#### 3.2.6. Reusability of TA-MAC.

**Fig 7:**
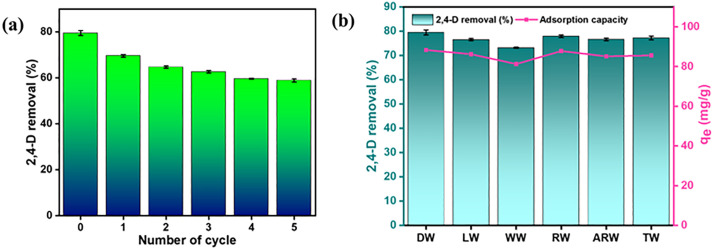
(a) Reusable capacity of TA-MAC (b) 2,4-D adsorption for diverse water sources using TA-MAC.

Reusable adsorbents are critical for achieving sustainable and effective water treatment. To determine their long-term viability, it is essential to evaluate the adsorbent’s performance through multiple use cycles under conditions that mimic a practical setting. For the reusability study, TA-MAC was successfully regenerated over five cycles using a 0.1M NaOH solution at an initial 2,4-D concentration of 50 mg/L. The highly basic conditions promote the deprotonation of 2,4-D, converting it to its anionic form. Simultaneously, the high pH creates negative charges on the TA-MAC surface via OH^-^ ion adsorption, causing repulsion of the anionic 2,4-D molecules and effectively desorbing them [60]. **Fig 7a** illustrates a minor reduction in 2,4-D adsorption removal efficiency with successive cycles. Even after five cycles of regeneration, the material’s removal efficiency remained strong, declining from 79.52 to 58.89%. This finding underscores the potential for TA-MAC to be an economically sound solution for water purification. Similar results were obtained using MAC derived from copper pod [22] and *Sargassum siliquastrum* [17]*.*

#### 3.2.7. Real water application.

The presence of various ions like phosphates, sulfates, nitrates, and chlorides can hinder the adsorption process by reducing the adsorbent capacity [17]. Real water samples are complex mixtures containing a variety of these ions. Therefore, to evaluate the practical utility of TA-MAC, its effectiveness in removing 2,4-D was tested using actual water sources. These sources included lake water (LW), well water (WW), river water (RW), agricultural runoff (AGW), and tap water (TW), as illustrated in **Fig 7b**. Distilled water, which served as the control, demonstrated the removal efficiency of 79.52% and an adsorption capacity of 88.36 mg/g. Significantly, the adsorption capacity stayed stable across all tested water samples, consistently achieving an adsorption capacity above 80 mg/g and a removal efficiency exceeding 70%. This demonstrates that the adsorption capacity was not substantially diminished relative to the distilled water baseline. This observation suggests that the complex matrix of real water, containing diverse ions, did not substantially compromise the overall adsorption effectiveness. Collectively, these results affirm the effectiveness of TA-MAC for removing 2,4-D in real water environments.

## 4. Conclusions

In the present study, magnetic activated carbon (TA-MAC) was prepared from *T. aurea* leaves, resulting in an irregular and well-developed porous structure and a large specific surface area. Morphological and magnetic characterization confirmed the successful incorporation of iron oxide nanoparticles onto the surface, indicating the formation of a superparamagnetic composite. The presence of a chlorine peak in the EDS spectrum after adsorption confirms the effective adsorption of 2,4-D onto TA-MAC. Changes observed in FTIR band intensities further suggest strong interactions between the adsorbent surface and 2,4-D molecules. The adsorption equilibrium data conformed to the Temkin isotherm model and were accurately expressed by pseudo-second-order kinetics. Combined FTIR and XPS analyses revealed that functional groups such as Fe-O, C-H, C-O, and O-H facilitate a multi-mechanistic adsorption path involving π-π interactions, hydrogen bonding, electrostatic binding, and pore filling. Furthermore, TA-MAC demonstrated robust reusability over five cycles and maintained consistent performance across diverse water samples, highlighting its potential for efficient 2,4-D remediation in practical wastewater treatment.

In summary, the synthesised TA-MAC demonstrates significant potential for practical water treatment applications owing to its high adsorption efficiency, magnetic recoverability, and reusability. The magnetic feature facilitates the easy separation of the adsorbent from the treated water, potentially diminishing operational complexity. Nonetheless, environmental factors and competing ions may affect adsorption efficacy in actual systems. Consequently, additional research on continuous-flow operation, long-term stability, and large-scale implementation is required.

## Supporting information

S1 Data Minimal Data Set 10.06.2026. (XLSX)
